# Parkinson Disease Recognition Using a Gamified Website: Machine Learning Development and Usability Study

**DOI:** 10.2196/49898

**Published:** 2023-09-29

**Authors:** Shubham Parab, Jerry Boster, Peter Washington

**Affiliations:** 1 University of Hawaii at Manoa Honolulu, HI United States; 2 Hawaii Parkinson Association Honolulu, HI United States; 3 Department of Information & Computer Sciences University of Hawaii at Manoa Honolulu, HI United States

**Keywords:** Parkinson disease, digital health, machine learning, remote screening, accessible screening

## Abstract

**Background:**

Parkinson disease (PD) affects millions globally, causing motor function impairments. Early detection is vital, and diverse data sources aid diagnosis. We focus on lower arm movements during keyboard and trackpad or touchscreen interactions, which serve as reliable indicators of PD. Previous works explore keyboard tapping and unstructured device monitoring; we attempt to further these works with structured tests taking into account 2D hand movement in addition to finger tapping. Our feasibility study uses keystroke and mouse movement data from a remotely conducted, structured, web-based test combined with self-reported PD status to create a predictive model for detecting the presence of PD.

**Objective:**

Analysis of finger tapping speed and accuracy through keyboard input and analysis of 2D hand movement through mouse input allowed differentiation between participants with and without PD. This comparative analysis enables us to establish clear distinctions between the two groups and explore the feasibility of using motor behavior to predict the presence of the disease.

**Methods:**

Participants were recruited via email by the Hawaii Parkinson Association (HPA) and directed to a web application for the tests. The 2023 HPA symposium was also used as a forum to recruit participants and spread information about our study. The application recorded participant demographics, including age, gender, and race, as well as PD status. We conducted a series of tests to assess finger tapping, using on-screen prompts to request key presses of constant and random keys. Response times, accuracy, and unintended movements resulting in accidental presses were recorded. Participants performed a hand movement test consisting of tracing straight and curved on-screen ribbons using a trackpad or mouse, allowing us to evaluate stability and precision of 2D hand movement. From this tracing, the test collected and stored insights concerning lower arm motor movement.

**Results:**

Our formative study included 31 participants, 18 without PD and 13 with PD, and analyzed their lower limb movement data collected from keyboards and computer mice. From the data set, we extracted 28 features and evaluated their significances using an extra tree classifier predictor. A random forest model was trained using the 6 most important features identified by the predictor. These selected features provided insights into precision and movement speed derived from keyboard tapping and mouse tracing tests. This final model achieved an average *F*_1_-score of 0.7311 (SD 0.1663) and an average accuracy of 0.7429 (SD 0.1400) over 20 runs for predicting the presence of PD.

**Conclusions:**

This preliminary feasibility study suggests the possibility of using technology-based limb movement data to predict the presence of PD, demonstrating the practicality of implementing this approach in a cost-effective and accessible manner. In addition, this study demonstrates that structured mouse movement tests can be used in combination with finger tapping to detect PD.

## Introduction

In the United States alone, Parkinson disease (PD) affects over 1 million individuals, with approximately 90,000 new diagnoses each year [1]. PD manifests with motor and nonmotor symptoms that impact the entire body, including challenges like micrographia that significantly disrupt daily life [2-6]. Unfortunately, symptomatic evaluation remains the sole diagnostic method for PD; an official diagnostic procedure is lacking. As a result, many cases go undiagnosed or misdiagnosed, hindering effective treatment [7-10]. Moreover, even unofficial PD diagnostic tests are costly, requiring specialized equipment and laboratory procedures [11-14]. Thus, there is an urgent need for scalable and accessible tools for PD detection and screening. Early diagnosis, which includes initiation of treatment and medication at an appropriate time, offers several benefits, including timely intervention and appropriate medication, leading to improved quality of life for patients [15,16].

PD affects limb movements, particularly lower hand movements, as evidenced by multiple studies [5,17-21]. Traditionally, PD diagnosis in the clinical setting relies on neurologists who consider medical history, conduct physical examinations, and observe motor movements and nonmotor symptoms [22,23]. Recently, researchers have explored the use of smartphones as a measurement tool for PD detection [24-26]. Previous studies have shown that PD can be detected by monitoring digital device activity, such as abnormal mouse movements and atypical typing patterns [27-33]. Building on these findings, our goal is to develop a user-friendly web application that offers a cost-effective and accessible diagnostic method, overcoming the limitations of in-person examinations and smartphone tests.

Previous studies investigating the use of finger movement for PD detection often faced challenges in accessibility due to their requirements for specialized equipment like accelerometers and gyroscopes [34,35]. For instance, Sieberts et al [31] used wearable sensors to gather accelerometer and gyroscope data, which might not be easily accessible to the general population. Chandrabhatla et al [36] discussed the transition from lab-based to remote digital PD data collection, but still relied on specific in-lab tools, which limited accessibility. Skaramagkas et al [37] used wearable sensors to distinguish tremors, while Schneider et al [38] found distinctive PD characteristics in shoulder shrugs, arm swings, tremors, and finger taps. Their findings emphasized arm swings and individual finger tremors as significant indicators.

Numerous studies have leveraged mobile apps, such as the work by Deng et al [32], which used the Mpower app to assess movement [33]. It is worth noting that older individuals, who are more vulnerable to PD, might not be as familiar with handheld devices like phones and tablets as they are with computers and laptops, which are more common among this age group [39-42]. Mobile phones became widely used in the early 2000s, with smartphones gaining popularity later, making them less familiar to older people [43-45]. On the other hand, many older adults have more experience with computers, which have been in use for a longer time [46]. This familiarity not only expands the potential participant group but also ensures more reliable data collection due to participants’ better understanding of the test procedures [47-49].

Keyboard typing’s potential for PD prediction has also been explored. In a study by Arroyo-Gallego et al [50], the neuroQWERTY method was used, which is based on computer algorithms that consider keystroke timing and subtle movements to detect PD. This approach was extended to uncontrolled at-home monitoring using participants’ natural typing and laptop interaction to detect signs of PD. The algorithm’s performance at home nearly matched its in-clinic efficacy. However, the lack of structure in this approach makes direct performance comparisons challenging. Additionally, Noyce et al [51] investigated genetic mutations and keyboard tapping over 3 years. They calculated risk scores using PD risk factors and early features. However, this study involved genetic information, which might not be accessible to many patients.

While drawing tests have been well studied, the investigation of mouse hovering to trace specific paths is limited. Rather than freehand tablet drawing, which is flexible but does not provide regulated data, Isenkul et al [52] used a tablet to help PD patients with micrographia. Yet since touchscreens are scarce on larger devices, a computer mouse provides a more accessible comparison [53,54].

Taking a different approach, researchers have used brain scans and biopsies to study changes in PD-related brain regions. Kordower et al [55] observed the progression of nigrostriatal degradation in PD patients over time. By analyzing brain regions, they found that a loss of dopamine markers 4 years after diagnosis was an indicator of PD.

These prior studies collectively contribute valuable insights into using digital devices for gathering motor-related PD data. Our research aims to expand upon this inspirational prior work by exploring the potential of an easily accessible web-based test involving keyboard finger tapping and mouse movements. We use a web application compatible with common devices to analyze these data, distinguishing individuals with and without PD to assess the feasibility of a more accessible and cost-effective detection method. While in-lab devices are precise but less accessible due to cost, web-based tests are cost-effective and accessible. Mobile apps are user-friendly but less familiar to older PD patients. Our web application, accessible on various devices, particularly computers, ensures familiarity and consistency and thus provides reliable data. Building on freeform drawing and keyboard tapping, our method adds structured tracing and key tapping tests, allowing direct performance comparisons.

We present an affordable and accessible method for PD detection using a web application that captures and analyzes lower hand movements during keyboard and mouse interactions. Keystrokes are measured by logging the time interval between prompts and keypresses, while false presses are recorded to detect finger shaking. Mouse movement is tracked every 500 milliseconds to assess precision and identify shaking or unintended movements. In a remote study with 31 participants, including 13 patients with PD and 18 controls without PD, we trained a machine learning (ML) model on 6 extracted movement features, achieving promising predictive performance. The model yielded an average *F*_1_-score of 0.7311 and an average accuracy of 0.7429. These results demonstrate the practicality of technology-driven limb movement data collection for effective PD detection.

## Methods

### Ethics Approval

This study obtained approval from the University of Hawaii at Manoa Institutional Review Board (IRB; protocol 2022-00857). Ensuring accurate identification of PD among participants was a significant challenge due to the lack of an official diagnostic certificate for PD. We therefore relied on self-report, and we required users to confirm their results with an intrusive dialogue that had to be dismissed before the test commenced, minimizing mistakes.

### Recruitment

Participants were recruited through the Hawaii Parkinson Association (HPA) and similar organizations. We collaborated with the former president of the HPA, who shared detailed information about our test via email. We set up a booth at the 2023 Hawai’i Parkinson’s Symposium, an event coordinated by the HPA. Attendees had the chance to take the test using a provided device and receive slips with the test URL. We welcomed participation from individuals both with and without PD who showed interest. Participants recruited by email were provided with a web application link to conveniently complete the tests remotely. The study included individuals with and without PD. This feasibility study consisted of a cohort of 31 participants. The age distribution was 65.226 (SD 10.832) years for all participants, 69 (SD 7.147) years for participants with PD, and 62.5 (SD 12.144) years for participants without PD.

Recognizing the limitations of our small sample size, we stress that this study represents an initial investigation into the utility of this test for PD detection. Our plan is to build upon these results through a larger-scale study involving a broader range of participants.

To address potential misclassification associated with using self-reporting, we implemented a comprehensive strategy. Participants were presented with an intrusive dialog box containing their entered demographics, including PD status, and were required to review and confirm its accuracy. They had the flexibility to modify their status and demographics, minimizing errors. Demographic data collection followed IRB guidelines with support from the HPA. Participants were provided the option to select “prefer not to answer” for certain demographic questions, encouraging test completion even without specific demographic details, as they were not essential to the study’s objectives.

### Parkinson Test

**Figure 1:**
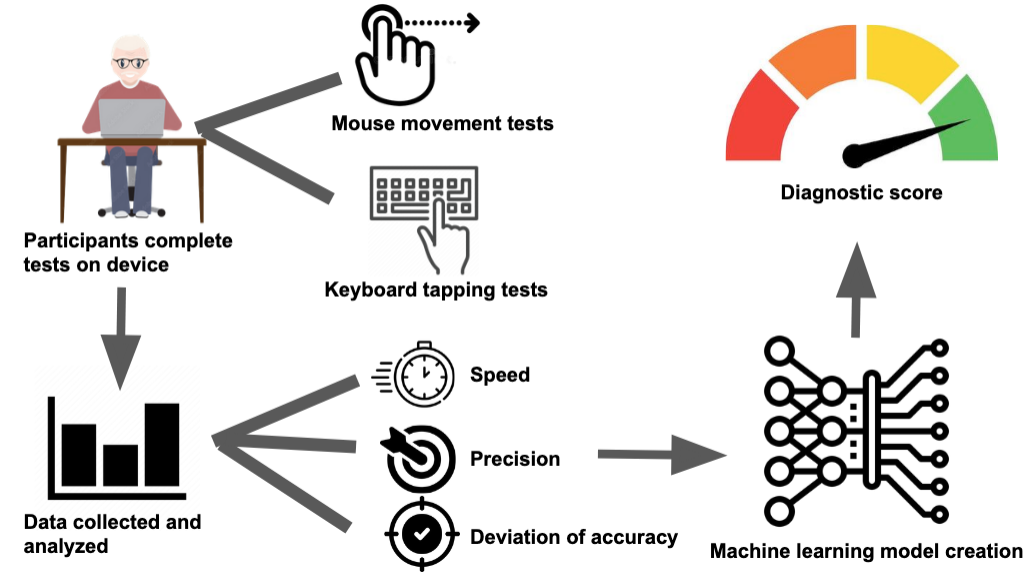
Overall study design. Participants completed mouse movement and keyboard tapping tests on their devices, from which data were collected and analyzed for speed, precision, and accuracy. A machine learning model was trained on these data to predict the presence of Parkinson disease.

**Figure 2:**
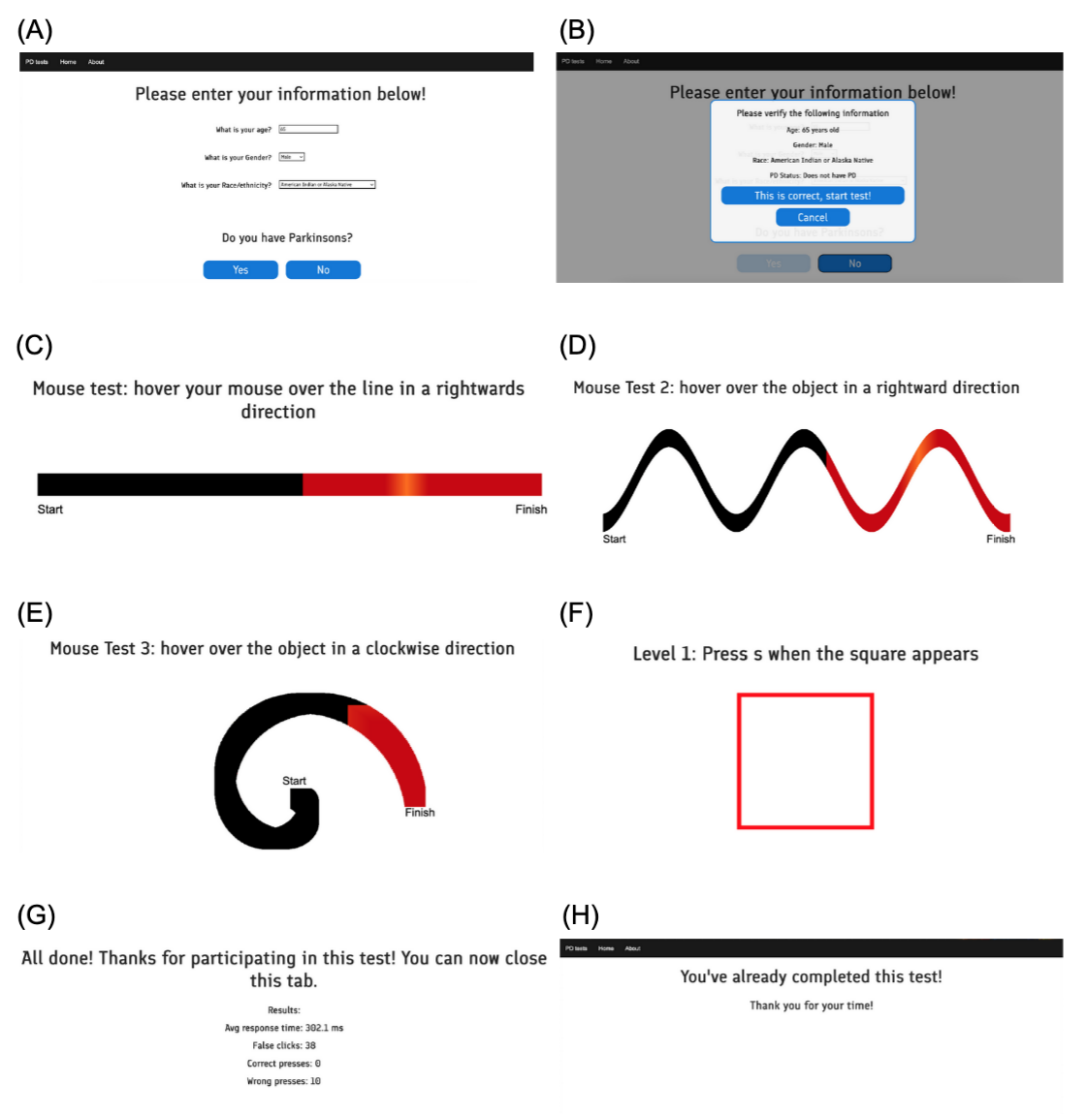
Screenshots of the web application. (A) Information is collected through a web-based form. (B) The participant is asked to confirm the entered information for correctness to prevent mistakes. (C) The linear mouse test asks users to trace the ribbon in a rightward direction. (D) The sine-wave mouse test asks users to trace the object. (E) The spiral mouse test asks users to trace a spiral. (F) The keyboard test asks users to press a certain letter when the red square prompt appears. (G) The test informs the participant that it is complete and thanks them for their time. (H) After completion, the test informs the user that they have already completed the test, minimizing duplicate entries.

Participants were instructed to type on a keyboard while the test recorded timestamps and finger movements corresponding to key positions (Figure 1). They pressed a specific key in response to on-screen signals, and we collected data on the expected key, pressed key, and response time. The measurement process had 3 increasing levels of difficulty with greater key randomization. The lowest difficulty of keyboard tapping prompted the press of a single key 10 times, while the second level alternated between 2 keys for 10 trials. The third difficulty changed the requested key to a random one for every press for 10 trials. For trackpad and mouse data, participants hovered the mouse along a designated path. This path was created in a way such that it took up certain percentages of the screen, as opposed to a set number of pixels, enabling it to adapt to the screen of the device being used and present an equal test to all participants. Starting with a straight line, subsequent levels introduced a sine wave–like shape and a spiral shape. Participants could monitor their progress by observing a highlighted portion of the shape, guided by animated direction indicators and “start” and “finish” markings. The interface of the web application, including the interfaces for demographic data collection and mouse and keyboard test administration, is show in Figure 2. We recorded data on position, time, and whether the mouse was inside or outside the indicated area. We also recorded the height and width of the participant’s device so it could be considered for calculations and could be used to recreate the user’s test.

We used a custom web application created with HTML, JavaScript, and CSS to collect data. For keyboard tapping, an HTML canvas was used to display a red square as a prompt. JavaScript was used to track keypress timing and calculate reaction times. For mouse tracing, an HTML canvas produced visuals of straight lines, sine waves, and spirals with direction indicator animations. JavaScript determined cursor location within the designated area and recorded mouse coordinates every 500 milliseconds.

After completing both tests, the collected data were securely transmitted and stored using a deta.sh base (Abstract Computing UG) facilitated by the deta.sh micros application programming interface, ensuring efficient data management.

This test was taken by participants both with and without PD who were primarily aged between 50 and 80 years (Table 1). The male to female ratio was nearly equal (Table 2), and the race of participants was predominantly White and Asian (Table 2). In addition, the ratio of participants with and without PD was slightly skewed toward those without PD (Table 2).

**Table 1:** Participant age distribution (N=31). The average age was 65.226 (SD 10.832) years for all participants, 69 (SD 7.147) years for participants with Parkinson disease, and 62.5 (SD 12.144) years for participants without Parkinson disease.

Age group (years)	Overall participants, n (%)	Participants with Parkinson disease, n (%)	Participants without Parkinson disease, n (%)
40-49	3 (10)	0 (0)	3 (10)
50-59	6 (19)	2 (6)	4 (13)
60-69	9 (29)	4 (13)	5 (16)
70-79	12 (39)	7 (23)	5 (16)
80-90	1 (3)	0 (0)	1 (3)

**Table 2:** Gender and race distribution. Gender was mostly balanced, with a male to female ratio of 15 to 16 and a total distribution slightly skewed toward female participants without Parkinson disease. Race was largely skewed toward White and Asian participants, with White and American Indian/Alaska Native participants having similar proportions of participants with and without Parkinson disease; Asian participants were mostly without Parkinson disease.

Characteristics				Participants (N=31), n (%)
**Participants with Parkinson disease**				
	**Gender**			
		Male	8 (26)	
		Female	5 (16)	
	**Race**			
		White	10 (32)	
		Asian	3 (10)	
		American Indian or Alaska Native	0 (0)	
		Unspecified	0 (0)	
**Participants without Parkinson disease**				
	**Gender**			
		Male	7 (23)	
		Female	11 (36)	
	**Race**			
		White	8 (26)	
		Asian	8 (26)	
		American Indian or Alaska Native	1 (3)	
		Unspecified	1 (3)	

### Statistical Analysis

We conducted a series of tests to measure keyboard keypresses, false presses, and timestamps as proxies for unintended movements, shaking, and anomalies. In the mouse hovering tests, we observed continuous mouse movement controlled by participants’ arms and recorded deviations from the centerline. These tests provided insights into events such as accidental deviations or vibrations, potentially indicative of PD symptoms. Additionally, we examined mouse data, focusing on hovering speed and precision as indicators of unintentional lower arm movements. We observed differences between participants with and without PD that could serve as potential disease indicators. These findings contribute to the advancement of PD detection methods by leveraging discernible distinctions between individuals with and without the condition.

During the test, we collected a total of 17 features in 4 major categories. One category assessed participant hand stability while tracing a straight line, considering mouse coordinates (x and y), deviation from the centerline, and whether the mouse was inside or outside the given region; this was measured every 500 milliseconds. Another category focused on tracing a curved line while maintaining hand stability, for which the coordinates (x and y) and whether the mouse was inside or outside the region were recorded. The third category measured response times and accuracy of key presses prompted by visual cues to evaluate reaction speed. The final category recorded the number of false presses during keyboard prompts as an indicator of unintended hand movements.

We extracted 28 features from each participant’s test. These included 17 baseline features along with additional features derived as a function of the baseline features. For instance, analyzing tracing deviation from the centerline produced multiple features, such as mean and maximum deviations. Incorporating screen width into time taken for tracing improved the feature’s association with PD, as seen in Table 3. An extra tree classifier predictor assessed the importance of each feature. We identified 6 key features as the most indicative of PD. These features were used to train a random forest model. These 6 features were selected due to their significantly higher importance scores compared to other related features as reported by the predictor. The selected features included mean deviation during straight line tracing, time to trace the sine wave relative to window width, spiral tracing time, average false presses, total response time for constant key tapping, and accuracy in responding to random key prompts.

Our random forest model was trained using an 80:20 train-test split. Due to the internal out-of-bag evaluation of random forest models, a separate validation set was not used. The model underwent 20 rounds of training with new train-test splits using an 80:20 ratio for each round. We used this evaluation method to account for our data set’s small size.

**Table 3:** Feature enhancement. A sample of features that were improved by considering other aspects of the data that affected them.

Feature	Enhancement	Original model *F*_1_-score (SD)	Model *F*_1_-score for enhanced feature (SD)	*F*_1_-score improvement
Amount of time taken for tracing straight line	Feature taken with respect to window width	0.6528 (0.1960)	0.7167 (0.1633)	0.0639
Number of correctly pressed keys when prompted with a random key	Feature taken with respect to average keypress time	0.6333 (0.1944)	0.6944 (0.2641)	0.0611

## Results

Our random forest ML model, trained on 6 features involving line tracing, sinusoid tracing, spiral tracing, accuracy and speed of keypress prompts, and false presses, yielded an average *F*_1_-score of 0.7311 (SD 0.1663). It also achieved an average accuracy of 0.7429 (SD 0.1400) (Table 4). All measurements were taken over 20 independent runs, with randomly sampled train-test splits created in each run. In addition, we trained the same set of 6 high-performing features on different types of ML models to determine the optimal model type (Table 5).

**Figure 3:**
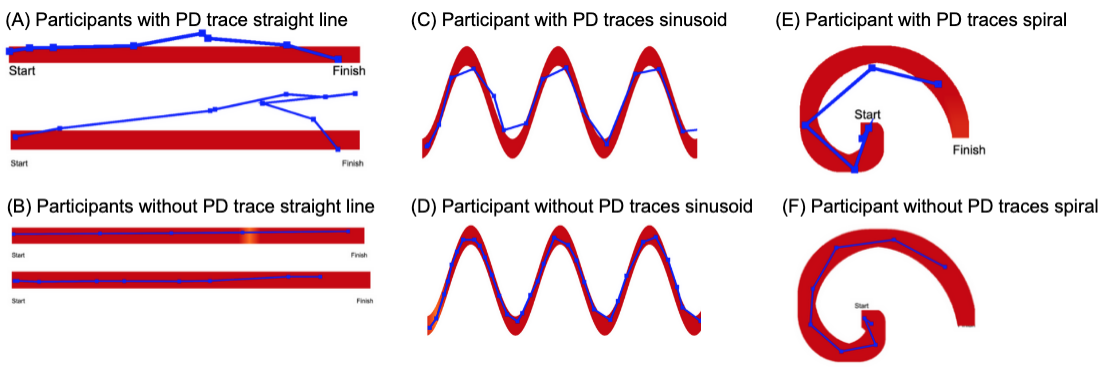
Sample traces of participants with and without PD. Generally, traces of participants with PD can be seen to be more irregular and less precise than those of participants without PD. PD: Parkinson disease.

We identified high-performing features and further analyzed their relationship with participants’ body movements. By reconstructing the traces based on the collected data, visual differences between participants with and without PD were observed. As shown in Figure 3, while most straight-line traces showed similarities, the traces of participants with PD exhibited sudden irregularities, whereas the traces of participants without PD had minimal irregularities. Additionally, significant differences were observed in sinusoid traces; participants with PD completed the test faster but with more irregularities and fewer points traced within the designated area. Conversely, participants without PD took more time but demonstrated greater precision. Similar patterns emerged in spiral traces, where participants with PD traced the spiral more rapidly but with less precision compared to participants without PD.

The performance of this model supports the feasibility of the automatic detection of PD through hand and finger movement analysis. These findings support the feasibility of using traced lines and curves as a potential method for predicting the presence of PD and other related conditions affecting limb movements using ubiquitous consumer devices such as laptops.

**Table 4:** Mean area under the curve, balanced accuracy, and *F*_1_-score of models trained on single and multiple features. Each individual feature was trained to evaluate its efficacy, shown in the first 28 content rows. An extra tree classifier was used to rank features by importance. The 6 most important features were used to train a final random forest model, shown in the last row.

Features used		Mean area under the curve (SD)	Mean balanced accuracy (SD)	Mean *F*_1_-score (SD)
**Models trained on single features**				
	Mean vertical deviation of tracing a straight line	0.6722 (0.232)	0.5 (0)	0.6722 (0)
	Maximum vertical deviation of tracing a straight line	0.6611 (0.257)	0.5 (0)	0.6611 (0)
	Net vertical deviation of tracing a straight line	0.6222 (0.1931)	0.55 (0.099)	0.6222 (0.2667)
	Total of the absolute values of vertical deviations of tracing a straight line	0.6138 (0.2328)	0.55 (0.099)	0.6138 (0.2667)
	Mean of the absolute values of vertical deviations of tracing a straight line	0.5861 (0.2057)	0.5 (0)	0.5861 (0)
	Amount of time taken for tracing straight line	0.6528 (0.1960)	0.6417 (0.2102)	0.6528 (0.3742)
	Amount of time taken for tracing straight line with respect to window width	0.7167 (0.1633)	0.5 (0)	0.7167 (0)
	Percentage of points traced in indicated width of a straight line	0.4056 (0.1196)	0.5 (0)	0.4056 (0)
	Number of points traced inside the expected width of a straight line (with no regard to time taken)	0.6319 (0.2393)	0.5667 (0.1307)	0.6319 (0.2696)
	Time taken to trace sine wave	0.7667 (0.1412)	0.65 (0.2118)	0.7667 (0.3277)
	Time taken to trace sine wave with respect to device window width	0.7472 (0.1753)	0.5 (0)	0.7473 (0)
	Percentage of traced points inside indicated sine curve	0.6444 (0.2516)	0.5 (0)	0.6444 (0)
	Number of points traced inside indicated sine curve with no regard to time taken	0.7 (0.2273)	0.6 (0.1409)	0.7 (0.2455)
	Time taken to trace a spiral	0.6986 (0.1586)	0.7583 (0.1633)	0.6986 (0.3363)
	Time taken to trace spiral with respect to device window width	0.7389 (0.2247)	0.5 (0)	0.7389 (0)
	Percentage of points traced inside the width of the indicated spiral	0.4181 (0.1816)	0.5 (0)	0.4181 (0)
	Number of points traced inside the width of the indicated spiral with no regard to time taken	0.7236 (0.1646)	0.6833 (0.1137)	0.7236 (0.2800)
	Total false key presses with single prompted key	0.5417 (0.0645)	0.5083 (0.0167)	0.5417 (0.16)
	Total false key presses with prompt key randomly chosen from 2 options	0.6 (0.2273)	0.6 (0.1225)	0.6 (0.2494)
	Total false key presses with prompt randomly chosen	0.2819 (0.1883)	0.375 (0.1581)	0.2819 (0)
	Total false key presses from all trials	0.6347 (0.2450)	0.575 (0.2048)	0.6347 (0.3309)
	Average response time when same key prompted	0.6194 (0.2208)	0.675 (0.1302)	0.6194 (0.2847)
	Average response time when semirandom (randomly chosen from 2 options) key prompted	0.6889 (0.2931)	0.5417 (0.1863)	0.6889 (0.1543)
	Average response time when random key prompted	0.6278 (0.2288)	0.725 (0.2)	0.6278 (0.2398)
	Number of correctly pressed keys (when prompted with same key) with respect to the average time taken	0.6445 (0.253)	0.5 (0)	0.6445 (0)
	Number of correctly pressed keys (with semirandom prompt) with respect to the average time taken	0.6833 (0.3313)	0.5 (0)	0.6833 (0)
	Number of correctly pressed keys when prompted with a random key	0.6333 (0.1944)	0.6333 (0.1944)	0.6333 (0.3997)
	Number of correctly pressed keys (when prompted with a random key) with respect to the average time taken	0.6944 (0.2641)	0.5 (0)	0.6944 (0)
**Model trained with 6 most important features**				
	Mean deviation from centerline when tracing straight line; amount of time taken to trace sine wave with respect to window width; amount of time taken to trace spiral; average false presses from 3 keyboard tapping trials; total response time taken when tapping constant (unchanging) key; number of correct keys with respect to average response time when prompted with random letter.	0.7311 (0.1663)	0.7429 (0.1400)	0.7311 (0.1663)

**Table 5:** Mean balanced accuracy and *F*_1_-score of different types of trained models. We trained several types of models on the same set of the 6 most important features and evaluated their average metrics over 20 runs. Between each run, the train-test split was resampled, maintaining the 80:20 ratio.

Model trained (20 runs)	Accuracy (SD)	*F*_1_-score (SD)
Random forest classifier	0.7429 (0.140)	0.7311 (0.1707)
Decision tree regression	0.5999 (0.1245)	0.5862 (0.1455)
Support vector classifier	0.6071 (0.1268)	0.5816 (0.1622)
Multilayer perceptron classifier	0.5214 (0.1707)	0.3740 (0.1939)

## Discussion

### Principal Results

This study provides evidence supporting the feasibility of remote collection of limb movement data using ubiquitously available consumer technology. We addressed concerns regarding device variations by considering device performance and specifications, such as screen height and width, which were measured and recorded by the application. Interestingly, excluding the time taken to complete the test improved the results for many extracted features. The findings suggest that either device performance affected the timing data or PD has limited influence on hand movement speed indicators such as key tapping or drawing. However, the latter scenario is unlikely, as multiple studies have shown that PD affects key tapping speed [56,57]. Additionally, false presses when prompted with a random key do not seem to be linked to PD, as this feature’s performance is notably weak. This might be due to displaying the next key before the prompt, leading to unintended key presses due to hand repositioning, a phenomenon common in older individuals regardless of PD status. Furthermore, our findings reinforce the use of measuring limb movements as an indicator of PD presence. The majority of models trained on individual extracted features yielded mean *F*_1_-scores and area under the curve (AUC) values surpassing 0.5, indicating a weak but existing correlation between the feature and the existence of PD. Additionally, 82% (23/28) of the features achieved an *F*_1_-score of at least 0.6, while 21% (6/28) achieved an *F*_1_-score of at least 0.7. Our final and most optimal model was able to achieve an accuracy of 74.29% and an *F*_1_-score of 73.11%. These results highlight a clear correlation between the speed and precision of tracing movements and the speed and accuracy of finger tapping with the presence of PD.

### Comparison to Previous Work

This study extends prior research by bringing lab-based movement testing to remote assessment on personal devices, enhancing accessibility and scalability. Unlike studies monitoring regular keyboard use, we used a structured test for better comparability. Additionally, our app includes structured tracing tests, exploring motor aspects other than keyboard tapping as PD indicators. Our model’s performance is comparable to the at-home neuroQWERTY test by Arroyo-Gallego et al [50], which achieved an AUC of 0.7311, while the clinic-based neuroQWERTY test achieved an AUC of 0.76. However, our model’s accuracy lags behind clinical tests with a similar aim, like that of Tsoulos et al [58], which achieved 93.11% PD detection accuracy, and that of Memedi et al [59], which reached 84% PD detection accuracy.

### Limitations

A key limitation of this study is the use of inconsistent devices. Using a specific device may introduce biases due to user unfamiliarity, whereas allowing participants to use their own devices may result in performance and specification variations. To address this, we standardized the collected data and recorded device aspects such as display width, height, and frames per second. This information enabled us to assess the influence of screen dimensions and device performance on user results. For example, by comparing the user’s mouse coordinates to the screen width in pixels, we determined the percentage of the screen that the mouse had moved, rather than the raw number of pixels, which would depend on the device used. However, other factors affecting the collected data, including differences between trackpads and mice, as well as keyboard types, have not been accounted for. These differences may have influenced our collected data and impacted our results. Additionally, the remote nature of the study posed a challenge, as participants completed it without supervision, potentially introducing errors and impacting results. It is worth noting that the ratio of participants with and without PD was 13 to 18, leading to an imbalance that could affect data analysis. Moreover, the study predominantly included participants of White and Asian ethnicities, introducing a racial imbalance that may impact the model’s accuracy for other racial groups if race influences the final prediction.

An additional limitation concerns the age difference in our sample. The participants without PD had an average age of 62.5 years, while the participants with PD had an average age of 69 years. Since we did not adjust for this age disparity, it may have influenced our results. In addition, the lack of an official PD diagnosis led us to rely solely on self-reports. Despite efforts to enhance accuracy, errors could have affected results. Furthermore, it is recognized that conditions like essential tremor (ET) cause symptoms similar to PD, potentially leading to misdiagnoses [60]. This scenario might have skewed our findings if individuals diagnosed with PD had ET. This concern could be addressed by inquiring about participants’ history of ET prior to the test and taking this into account for analysis.

### Further Research

ML holds promise for predicting movement-related conditions, including ET, and its application can be extended to other movement-impacting diseases. Standardizing a comprehensive test could offer individuals a single, straightforward assessment to evaluate their likelihood of having different health conditions. By incorporating diverse shapes for tracing, such as those involving sudden stops or changes in direction, additional valuable insights into hand movements of participants both with and without PD can be gleaned. To address limitations associated with unsupervised remote studies, a supervised approach with real-time monitoring could be implemented, providing immediate feedback to ensure protocol adherence and improve data reliability. Additionally, collecting information on participants’ device types can help address potential bias arising from device disparities. With a sufficiently large sample size, subgroup analysis based on device type could mitigate the impact of device variations on data and strengthen the validity of the findings. Some related studies have used significantly more participants [58,59]. Expanding the participant sample size would support the generalizability of our findings.

Another potentially fruitful avenue of expanding PD screening tools would be to include additional data modalities such as computer vision. Computer vision analysis has been successfully used for a variety of health screening and diagnostic tasks, including abnormal hand movements and movement of other body parts for conditions such as autism [61-67]. Using such techniques for PD screening can expand the performance of the tools through a more comprehensive and multimodal analysis.

## Abbreviations

AUC: area under the curve
ET: essential tremor
HPA: Hawaii Parkinson Association
IRB: institutional review board
ML: machine learning
PD: Parkinson disease

## Data Availability

The data sets generated and analyzed during this study have been deidentified and are available in the GitHub repository [68].
